# Treatment Access and Caregiver Experience in Pediatric Rhabdomyosarcoma: Results of an Online Survey

**DOI:** 10.3390/children12040435

**Published:** 2025-03-29

**Authors:** Jamil Almohtasib, Timothy C. Boswell, Candace F. Granberg, Patricio C. Gargollo

**Affiliations:** 1 Department of Urology, Mayo Clinic, 200 First St. SW, Rochester, MN 55905, USA; almohtasib.jamil@mayo.edu (J.A.);; 2 Department of Urology, Children’s of Alabama, University of Alabama at Birmingham, 1600 7th Avenue South, Lowder Suite 318, Birmingham, AL 35233, USA

**Keywords:** pediatric cancer, rhabdomyosarcoma, treatment access, caregiver experiences

## Abstract

Background/Objectives: Pediatric rhabdomyosarcoma is a rare and poorly understood disease. Patients and parents can have varying experiences including barriers to care, time to treatment, and treatments offered. Here, we report on patient experiences regarding their care of pediatric rhabdomyosarcoma. Methods: Two separate online parent support groups were invited to participate in a survey. The survey included questions that sought to collect patient demographics, history of rhabdomyosarcoma, treatment timelines, and barriers to care. Results: A total of 215 surveys were completed. The average time from diagnosis to treatment was 12 days (SD = 14). Only 26% were offered fertility preservation prior to treatment. For patients with recurrence, an average of 75 days passed between detection of recurrence and treatment re-initiation. Patients traveled to centers with a dedicated sarcoma program in 52% of the cases. A total of 42% of parents sought a second opinion. Of those, the majority had to wait between one week and one month to be seen by another expert. Conclusions: The data collected from the survey suggests there are several opportunities to improve care among patients with pediatric rhabdomyosarcoma. Many patients may benefit from more efficient rhabdomyosarcoma referral networks, delivering patients to experts who can quickly begin multidisciplinary treatment.

## 1. Introduction

Although pediatric rhabdomyosarcoma (pRMS) is the most common soft tissue sarcoma in children, it remains a rare tumor, with approximately 4.5 cases per 100,000 diagnosed per year in the United States [1]. While significant progress has been made in the treatment and outcomes of patients with these tumors, little is known about caregiver experiences during the treatment process. Understanding parental recall of treatment details, the second-opinion process, and access to fertility preservation is essential for identifying gaps in care and improving patient outcomes.

Caregivers of children with cancer experience significant emotional and psychological distress, often facing challenges related to navigating the healthcare system, the financial burden, and decision making regarding treatment options [2,3]. These challenges can have long-term impacts on both caregivers and patients, affecting overall well-being and their treatment journey [4]. Additionally, patients with rare cancers like pRMS often face difficulties in receiving timely and effective care due to the rarity and complexity of their conditions [5,6].

To this end, we created an anonymous online questionnaire for parents of children diagnosed with pRMS. Our study aims to evaluate treatment access and caregiver experiences, including parental recollection of tumor site, stage, treatment, and relapse; assess delays in treatment initiation at diagnosis and recurrence; analyze the second-opinion process and potential barriers; and examine parents’ access to fertility preservation and their willingness to donate their child’s tissue for medical research.

We hypothesize that parents of children with pRMS have an excellent recollection of their child’s diagnosis and treatment and that treatment initiation delays exist both at primary diagnosis and relapse. We further hypothesize that parents face barriers in obtaining second opinions.

## 2. Materials and Methods

After consultation with our institution’s survey research center, we designed a 39-item online survey. Questions were divided into the following sections: initial diagnosis, treatment, patient outcomes, second opinions, fertility preservation, and tissue donation. Initial diagnosis section included the age at diagnosis, gender, the primary site, histology, how was tumor tissue obtained, lymph node involvement and metastasis at diagnosis—and if there was metastasis, how many other sites the tumor spread to—whether there was bone marrow involvement, the presence of chromosomal translocation, and FOX01 fusion status. The treatment section involved questions regarding the modality of initial therapy (chemotherapy, radiation, surgery, or multimodal) and number of days from diagnosis to treatment initiation. Patient outcomes included the outcome after initial treatment (disease-free vs. partial remission) and whether recurrence occurred and its type (local recurrence vs. metastatic). The following section assessed whether caregivers sought second opinions or not, time needed to receive second-opinion recommendations, and whether they were charged for receiving second opinions. Caregivers were also asked whether they had to travel more than 100 miles to receive expert care, which was defined as any center that has a multidisciplinary sarcoma team and should include the following: a surgeon experienced in treating sarcoma, medical oncologist, radiologist, and histopathologist with access to molecular pathology [7]. Further sections assessed whether caregivers were offered fertility preservation for their children, and their willingness to donate tissue for pRMS research.

Members of two online support groups were invited to participate in our study between February and March 2019. One group was for parents of children living with pRMS and the other was for parents of deceased children from pRMS. All surveys were anonymous and no protected health information was collected. All respondents who were caring for or had cared for a patient with pRMS were included. Multiple responses from the same IP address or incomplete surveys were excluded.

This study was conducted in accordance with the Declaration of Helsinki. Given that the study was based on an anonymous online survey with no collection of identifiable information, it was exempt from institutional review board (IRB) at our institution. Participants were informed about the study and its objectives through a brief text introduction. Consent was implied through the voluntary completion of the survey.

Descriptive statistics were used to summarize survey results; categorical variables are presented as percentages, while numerical variables are presented as mean and standard deviation. Patient and disease characteristics as well as treatment modalities and outcomes were tested for associations with traveling for care and seeking a second opinion using Chi-square test and student’s *t*-test. Incomplete survey responses were excluded to maintain data integrity. For individual missing responses within completed surveys, no imputation methods were applied; only available data were analyzed. All analyses were performed using the SAS software package version 9.4 (SAS Institute, Inc. Cary, NC, USA).

## 3. Results

In total, 215 surveys were completed from unique IP addresses. Patient demographics and cancer characteristics are shown in Table 1. The mean age at diagnosis was 7.7 years (SD 5.2) and 54% of patients were male. The median year of diagnosis was 2016 (IQR: 2014–2018) and only 8% of patients were diagnosed before 2010. Histology was embryonal in 55% of cases, alveolar in 38%, spindle cell in 3%, botryoid in 3%, and other in the remainder. The most common sites were genitourinary (35%), head and neck (14%), parameningeal (14%), and musculoskeletal (13%). Initial biopsy was most commonly obtained through open surgery (52%), followed by needle biopsy (27%) and endoscopic or laparoscopic biopsy (21%). A total of 32% of parents reported their child having a positive lymph node at diagnosis and 40% reported metastatic disease at initial diagnosis, with 32% of those with one metastatic site and the remainder with multiple metastatic sites. Relatedly, chemotherapy alone was the initial treatment in 68% of patients; 14% had surgery alone. The remainder of patients (18%) had multimodal therapy, which was defined as receiving a combination of chemotherapy and surgery and/or radiation. Of note, of the 68 parents reporting surgery as part of their child’s initial treatment, only 46% reported this being a complete resection. At the conclusion of the initial therapy, 81% reported being told that their child was disease-free and 13% that they were in partial remission. A total of 26% developed disease recurrence, with half being local recurrence and half metastatic recurrence.

**Figure 1:**
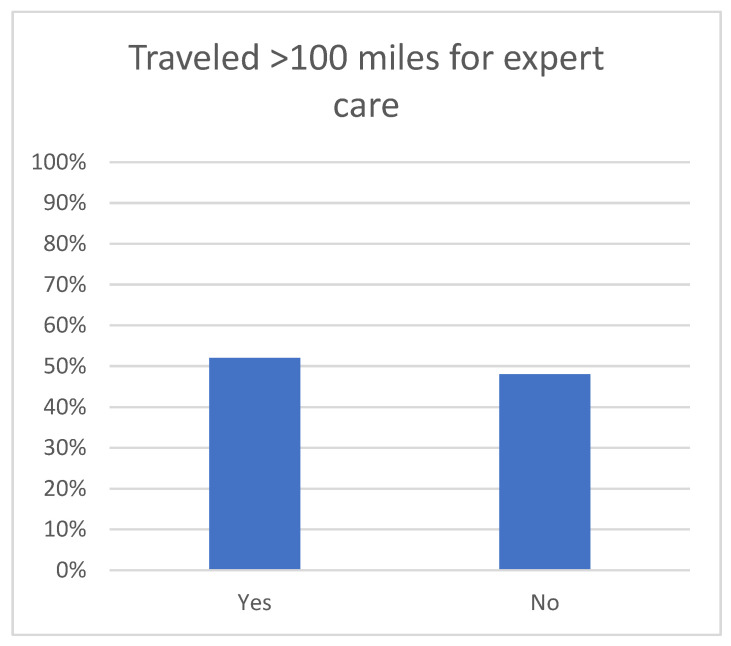
A total of 52% of families traveled more than 100 miles to seek expert pRMS care.

**Figure 2:**
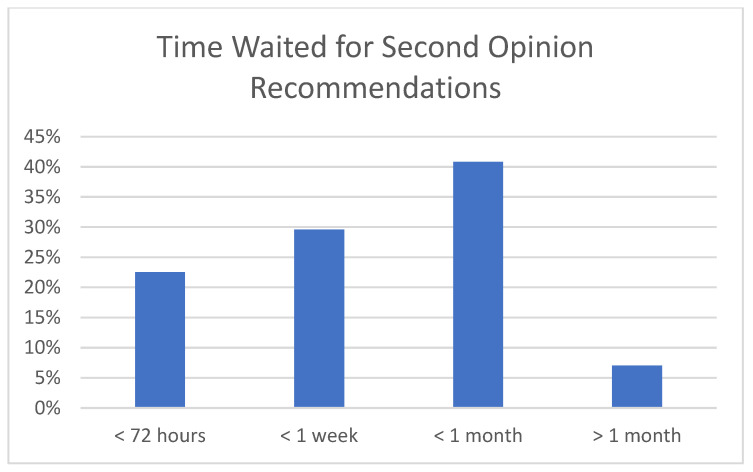
This figure demonstrates the amount of time waited by families to meet with the provider offering them a second opinion.

**Figure 3:**
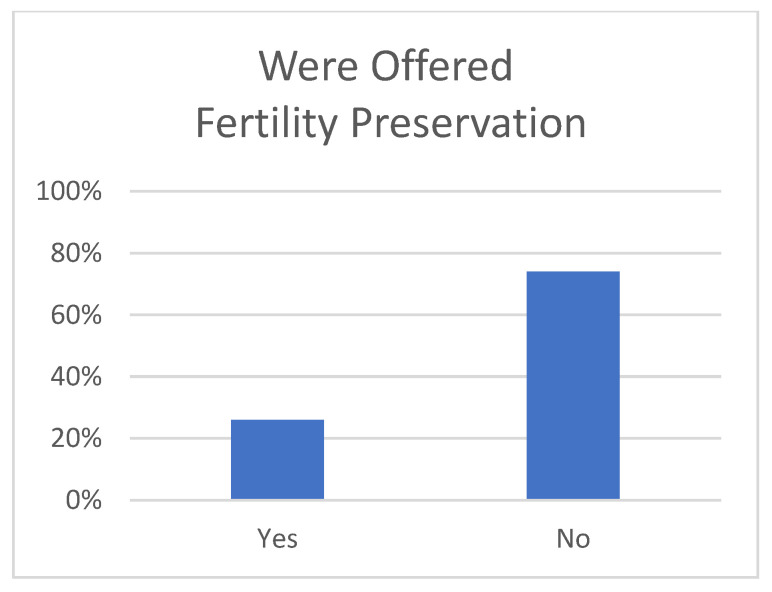
Fertility preservation was offered to 26% of pRMS patients’ parents.

For access to care and timing of care, 52% reporting traveling more than 100 miles for expert care (Figure 1). The mean number of days from diagnosis to initiation of treatment was 12 days (SD 14), and 22% had their treatment initiated more than 2 weeks after diagnosis. A total of 42% reported seeking a second opinion. Of those seeking a second opinion, the time to receiving second opinion recommendations was between one week and one month in 41% and longer than one month in 7% (Figure 2). Of those seeking a second opinion, 46% reported being charged for it. Several respondents wrote in describing the means of seeking a second opinion. These varied from recommendations from their primary oncologist, personal research online, recommendations from Facebook support groups, and personal contacts through physician friends. Notably, none referenced any formal process or rhabdomyosarcoma referral network. Only 23% reported being offered fertility preservation prior to initiating treatment (Figure 3), while 92% reported that they would have considered tissue donation if offered it. For the 26% who developed disease recurrence after an initial treatment course, an average of 75 days passed between detection of recurrence and initiation of treatment for recurrence.

**Figure 4:**
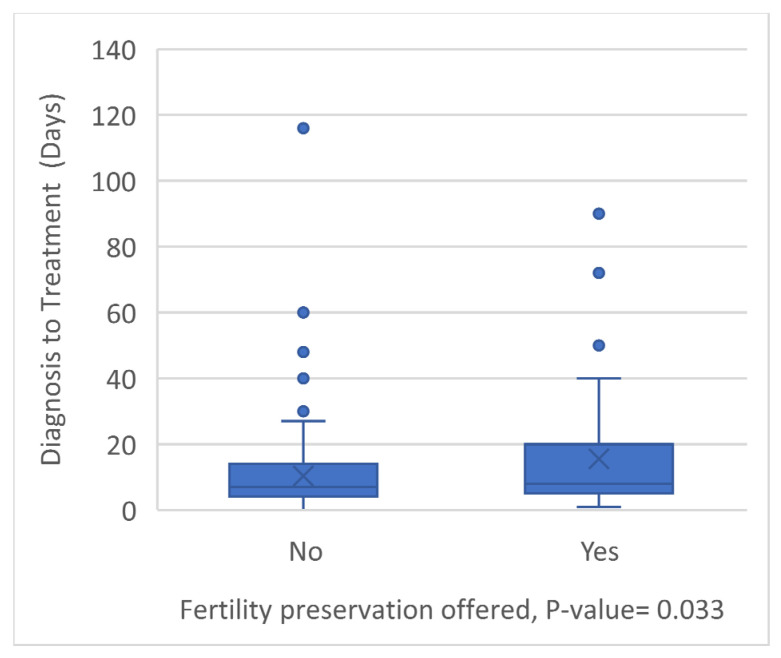
A boxplot demonstrating the difference in Dx to Rx days between patients offered fertility preservation.

When testing for associations, there was no association between cancer histology, site, extent of disease, treatment modality, or treatment outcome and whether patients traveled for care or sought a second opinion. Likewise, traveling for care and seeking a second opinion were not associated with being offered fertility preservation. However, patients who were offered fertility preservation had a longer duration between diagnosis and initiation of treatment (15.5 ± 18.2) compared to those who were not offered it (10.33 ± 12.7) (*p*-value = 0.033) (Figure 4).

When answering this final question, “What was one thing you wish you knew back when all of this started that you know now?”, here are some of the notable responses:

− “How many delays there would be”
− “That our HMO would provide care outside of our institution, due the rarity of her diagnosis and lack of familiarity with her disease among local clinicians”
− “To preserve her reproductive organs”
− “Go to a Sarcoma center. Send tissue off to a Rhabdo specialist.”
− “Do your research! Get a second or even third opinion!”
− “I wish I had known about rhabdo specialists.”
− “It’s an art, not a science.”
− “I wish we would have tried resection when it came back. It’s like the docs gave up. That was never offered as an option.”
− “The pediatric rhabdo Facebook group”
− “The different treatment options available abroad”
− “Ask questions and do not assume that the doctors are doing everything possible to save the patient. The earlier you act, the better. Seek a second opinion.”
− “Push the pediatrician! She put us off for months and my daughter was stage 3 when she could have been treated much earlier.”
− “Insist surgery and radiation—more aggressive treatment.”
− “I wish I would have known about fertility preservation.”
− “Go to the right doctor immediately and push doctors to be more aggressive and quicker in treatments! It’s such an aggressive and quick cancer and some doctors drag their feet too much.”
− “Don’t listen blindly to the health care professionals. Ask questions and engage in discussions to understand better the decisions being taken, their consequences, and the options available.”
− “I would have traveled for a second opinion.”
− “Make sure you are talking with a sarcoma specialist.”
− “I wish I would have done more research and been a better advocate for my son.”

## 4. Discussion

Herein, our survey of 215 parents of children treated for rhabdomyosarcoma demonstrated that only about half traveled to seek expert care and 42% sought a second opinion. Treatment delays were experienced by many, especially those seeking a second opinion and seeking treatment for a recurrence. Furthermore, fertility preservation was offered in a minority and most parents reported that they would have been willing to donate tissue for research purposes if asked. All of these things suggest room for improvement in pRMS care.

Treatment delays appear to be common among children with RMS. Diagnosis delays have been well studied in pediatric solid tumors, especially in developing countries [8,9,10]. Patient age, parental education, socioeconomic status, rural residence, specialty of the first assessing physician, and cancer site (genitourinary especially) all carried associations to delays. Because rhabdomyosarcoma can be a rapidly progressive disease, any delay in diagnosis or treatment can have a profound impact on outcomes. Our results have showed an increased delay in patients who were offered fertility preservation. The delay in the initiation of treatment for cancer patients in association with fertility preservation has been well described in the literature, and the risk of delaying therapy might outweigh the benefits of fertility preservation in highly aggressive tumors such as rhabdomyosarcoma [11,12]. Newer techniques in fertility preservation have been described and found to have no significant delay on treatment; the implication of those methods would benefit patients with pRMS [13]. These facts suggest the need to develop systems for prompt diagnosis followed by efficient delivery of care. However, the rapidity of care is not the only focus, but instead, patients need expert care for this uncommon group of diseases. In fact, a large proportion of the free-text responses from pRMS parents focused on these two facts: They wished care had moved forward more swiftly, but they also wished they had searched out more expert and thorough care.

Therefore, a brief delay in order to receive a higher level of care would conceivably be a reasonable trade-off. About 42% of our cohort reported seeking a second opinion, and 48% of those had to wait longer than a week to meet with the provider. In pediatric oncology, parents tend to seek a second opinions for confirmation about the treatment protocol initially proposed, and parents with higher socioeconomic status and educational level are more likely to seek a second opinion [14,15]. The rarity of the diagnosis and our survey results suggest that access to expert care is somewhat limited. Barriers to expert care include insurance coverage, social situation, travel expenses, and concern that doing so will disrupt the physician/patient relationship by sending a message of distrust [16]. To the last point, though, surveyed physicians who care for cancer patients reported a generally positive perspective towards second opinions, saying that they can enhance patient satisfaction and overall care [17].

Sadly, based on our survey, few children with RMS (26%) are being offered fertility preservation. It is increasingly evident that parents, current patients, and cancer survivors value fertility preservation as part of their treatment plan and that the loss of fertility causes significant distress [18,19]. Furthermore, multiple national and international professional organizations have called for patient education and guidance regarding fertility preservation before the initiation of gonadotoxic therapy [20]. In spite of this, several studies have confirmed that even post-pubertal cancer patients are rarely evaluated for, counseled towards, or actually undergo fertility preserving procedures [21]. In one study, only 19% of cancer survivors were offered fertility counseling [22]. In pediatric populations, the uptake of FP remains low; a study reporting data on 61 parents and children showed that 84% of high-risk patients were not counseled on fertility preservation [23]. Multiple barriers have been identified including cost, patients’ health beliefs and health literacy, clinicians’ approaches and skills, and facility resources [24,25,26,27,28]. The mean age of our cohort was 7.7 years, which presents unique challenges in FP discussions. Prepubertal children, particularly males, often cannot provide sperm samples, and FP techniques in this group remain largely experimental. This uncertainty may deter clinicians from delaying urgent cancer treatments to pursue FP methods with unproven efficacy. Patient decision aids have been shown to significantly increase fertility preservation knowledge and decrease decisional conflict [29]. This needs to be improved, even in the pediatric oncology population, because, while pre-pubertal tissue cryopreservation is experimental, it has led to the successful repopulation of ovarian tissue and function and even live birth [30,31,32].

Our surveyed parents demonstrated that they are very willing to consider tissue donation for research. One of the fundamental limitations to scientific breakthroughs in pRMS is the relative scarcity of tissue for study. Small-volume biopsies are the most common means of initial diagnosis, and a sizeable percentage of treatment protocols do not include surgical resection. Therefore, physicians should consider having discussions of post-mortem donation in the event that cure is unlikely. In cases of bereaved tissue and organ donation, there are some clues from the trauma and organ donation literature that parents’ decision making is positively impacted when their concerns about the end-of-life process are addressed, they experience empathy in the process, they are educated on the option, and they have overall satisfaction with the healthcare experience [33,34,35]. Another important aspect in obtaining post-mortem tumor tissue for study is doing so before tissue degradation via rapid autopsy. However, the required rapidity can generate pressure on a mourning family at a delicate time. On this topic, addressing the possibility of rapid autopsy well in advance appears to be a key aspect of the physician approach, as evidenced from the breast cancer literature: 87% of patients with metastatic breast cancer reported a willingness to donate tissue at death, but many noted that it is important to them that doctors ask about rapid autopsy at early-stage disease rather than late-stage disease [36]. Parents seem to be eager to aid pRMS research, expressing a willingness to donate tissue from their own children, but education, timing, and empathy must guide the process.

Our study highlights several strategies of improvement that can be carried out in pRMS care. Establishing strong and well-designed referral networks to ensure timely access to specialized sarcoma centers is essential. Delays in admission and treatment initiation have been linked to poorer outcomes in pediatric patients with advanced-stage disease. Despite guidelines recommending discussions about fertility risks and preservation options prior to treatment, implementation remains inconsistent [37]. Integrating routine fertility preservation discussions into patient care protocols can address this gap. Finally, fostering early and compassionate conversations about post-mortem tissue donation may advance research efforts. While specific data on parental willingness in pRMS are limited, early discussions about tissue donation have been shown to increase participation and facilitate critical discoveries in pediatric oncology research.

Despite the valuable insights gained from this study, several limitations must be acknowledged. Firstly, as a survey-based study, our findings are subject to recall bias. Caregivers may not have precisely remembered details regarding treatment timelines and clinical information like cancer histology. Potentially introducing inaccuracies in reporting, this is particularly relevant for families whose children were diagnosed several years ago. Additionally, our study is limited by a relatively small sample size. While our sample provides important perspectives on caregiver experiences, the results may not fully represent the broader population of families affected by pediatric rhabdomyosarcoma, particularly those who do not participate in online support groups.

We have also implemented a response validation measure by excluding multiple responses from the same IP address to minimize duplicate participation. However, this approach may have excluded valid responses from different caregivers within the same household. For example, two family members with differing perspectives on the caregiving experience may have attempted to complete the survey but were restricted due to this exclusion criterion. Finally, our survey did not assess the psychosocial impact on caregivers. The emotional and mental burden of navigating a child’s cancer diagnosis and treatment is a crucial component of the caregiver experience. Future studies should aim to incorporate validated measures of caregiver distress and anxiety, to better understand the full scope of challenges faced by families. Despite these limitations, this survey demonstrates that there is significant room for improvement in pRMS care, particularly as it relates to access to expert pRMS care, offerings of fertility preservation, and the collection of RMS tissue for further research.

## 5. Conclusions

Our study points out critical gaps in pediatric rhabdomyosarcoma care, including delays in treatment initiation, limited access to expert centers, and the under-usage of fertility preservation. Despite 42% of caregivers seeking second opinions, many faced significant wait times, and only 26% reported being offered fertility preservation for their child. Additionally, while families demonstrated a strong willingness to contribute to research through tissue donation, structured referral networks and improved education on available resources remain lacking. These findings emphasize the need for more streamlined care pathways, increased awareness of fertility preservation, and better support systems for caregivers navigating this challenging journey.

## Figures and Tables

**Table 1:** Baseline patient characteristics as well as types and extent of their rhabdomyosarcoma at diagnosis.

	Total (N = 215)
AGE	
N	215
Mean (SD)	7.7 (5.2)
GENDER	
Male	115 (53.5%)
Female	100 (46.5%)
SITE	
Adrenal	1 (0.5%)
Diaphragm	2 (0.9%)
Genitourinary (bladder and/or prostate)	33 (15.3%)
Genitourinary (not including above)	41 (19.1%)
Head and Neck (Non-parameningeal)	31 (14.4%)
Hepatobiliary	4 (1.9%)
Limbs	44 (20.5%)
Lungs	1 (0.5%)
Nasopharynx	1 (0.5%)
Orbit (eye and surrounding structures)	20 (9.3%)
Parameningeal	30 (14.0%)
Primary unknown	2 (0.9%)
Spine	3 (1.4%)
Trunk	2 (0.9%)
HISTOLOGY	
Alveolar	80 (38.3%)
Anaplastic	1 (0.5%)
Botryoid	5 (2.4%)
Embryonal	114 (54.5%)
Pleomorphic	2 (1.0%)
Spindle cell	7 (3.3%)
Missing	6
POSITIVE LYMPH NODES AT DIAGNOSIS	
No	140 (68.3%)
Yes	65 (31.7%)
Missing	10
METASTASIS AT DIAGNOSIS	
No	127 (60.5%)
Yes	83 (39.5%)
Missing	5

## Data Availability

The datasets generated and analyzed during the current study are available from the corresponding author upon reasonable request.

## Abbreviation

The following abbreviations are used in this manuscript:

pRMS	Pediatric rhabdomyosarcoma

## References

[1] Gloeckler Ries L.A., Reichman M.E., Lewis D.R., Hankey B.F., Edwards B.K. (2003). Cancer survival and incidence from the Surveillance, Epidemiology, and End Results (SEER) Program. Oncologist.

[2] Effendy C., Uligraff D.K., Sari S.H., Angraini F., Chandra L. (2022). Experiences of family caregivers of children with cancer while receiving home-based pediatric palliative care in Indonesia: A qualitative study. BMC Palliat. Care.

[3] Ismael N., Jaber A., Malkawi S., Al Awady S., Ismael T. (2024). Exploring coping strategies among caregivers of children who have survived paediatric cancer in Jordan. BMJ Paediatr. Open.

[4] Olsavsky A.L., Sutherland-Foggio M., Stanek C.J., Hill K.N., Himelhoch A.C., Kenney A.E., Humphrey L., Olshefski R., Skeens M.A., Nahata L. (2024). Factors associated with caregiver strain among mothers and fathers of children with advanced cancer. Palliat. Support. Care.

[5] Papyan R., Tamamyan G., Danielyan S., Tananyan A., Muradyan A., Saab R. (2019). Identifying barriers to treatment of childhood rhabdomyosarcoma in resource-limited settings: A literature review. Pediatr. Blood Cancer.

[6] Kostadinov K., Iskrov G., Musurlieva N., Stefanov R. (2025). An Evaluation of Rare Cancer Policies in Europe: A Survey Among Healthcare Providers. Cancers.

[7] Wilson R., Reinke D., van Oortmerssen G., Gonzato O., Ott G., Raut C.P., Guadagnolo B.A., Haas R.L.M., Trent J., Jones R. (2024). What Is a Sarcoma ‘Specialist Center’? Multidisciplinary Research Finds an Answer. Cancers.

[8] Sari N.M., Devansyah S., Modjaningrat I., Suryawan N., Susanah S., Rakhmillah L., Wahyudi K., Kaspers G.J.L. (2023). Type of cancer and complementary and alternative medicine are determinant factors for the patient delay experienced by children with cancer: A study in West Java, Indonesia. Pediatr. Blood Cancer.

[9] Winestone L.E., Wilkes J.J., Puccetti D., Keegan T.H.M., Henk H.J., McPheeters J., Kahn J.M., Ginsberg J., Wong S., Timberline S. (2024). Time to diagnosis among young patients with cancer. Pediatr. Blood Cancer.

[10] Lombe D.C., Mwamba M., Msadabwe S., Bond V., Simwinga M., Ssemata A.S., Muhumuza R., Seeley J., Mwaka A.D., Aggarwal A. (2023). Delays in seeking, reaching and access to quality cancer care in sub-Saharan Africa: A systematic review. BMJ Open.

[11] Kappy M., Lieman H.J., Pollack S., Buyuk E. (2021). Fertility preservation for cancer patients: Treatment gaps and considerations in patients’ choices. Arch. Gynecol. Obstet..

[12] Meernik C., Engel S.M., Baggett C.D., Wardell A., Zhou X., Ruddy K.J., Wantman E., Baker V.L., Luke B., Mersereau J.E. (2023). Time to cancer treatment and reproductive outcomes after fertility preservation among adolescent and young adult women with cancer. Cancer.

[13] Doungkamchan C., Orwig K.E. (2021). Recent advances: Fertility preservation and fertility restoration options for males and females. Fac. Rev..

[14] Mordechai O., Tamir S., Weyl-Ben-Arush M. (2015). Seeking a second opinion in pediatric oncology. Pediatr. Hematol. Oncol..

[15] Fuchs T., Hanaya H., Seilacher E., Koester M.-J., Keinki C., Liebl P., Huebner J. (2017). Information Deficits and Second Opinion Seeking—A Survey on Cancer Patients. Cancer Investig..

[16] Peier-Ruser K.S., von Greyerz S. (2018). Why Do Cancer Patients Have Difficulties Evaluating the Need for a Second Opinion and What Is Needed to Lower the Barrier? A Qualitative Study. Oncol. Res. Treat..

[17] Shin D.W., Cho J., Yang H.K., Kim S.Y., Mok H.K., Lee H., Park S.M., Huh J.S., Ryu J., Park J.H. (2016). Attitudes towards second opinion services in cancer care: A nationwide survey of oncologists in Korea. Ultrasound Med. Biol..

[18] Logan S., Anazodo A. (2019). The psychological importance of fertility preservation counseling and support for cancer patients. Acta Obstet. Gynecol. Scand..

[19] Rodriguez-Wallberg K.A., Ahlgren J., Smedby K.E., Gorman J.R., Hellman K., Henriksson R., Ståhl O., Wettergren L., Lampic C. (2023). Prevalence and predictors for fertility-related distress among 1010 young adults 1.5 years following cancer diagnosis—Results from the population-based Fex-Can Cohort study. Acta Oncol..

[20] Yang E.H., Strohl H.B., Su H.I. (2024). Fertility preservation before and after cancer treatment in children, adolescents, and young adults. Cancer.

[21] Selter J., Huang Y., Becht L.C.G., Palmerola K.L., Williams S.Z., Forman E., Ananth C.V., Hur C., Neugut A.I., Hershman D.L. (2019). Use of fertility preservation services in female reproductive-aged cancer patients. Am. J. Obstet. Gynecol..

[22] Young K., Shliakhtsitsava K., Natarajan L., Myers E., Dietz A.C., Gorman J.R., Martínez M.E., Whitcomb B.W., Su H.I. (2019). Fertility counseling before cancer treatment and subsequent reproductive concerns among female adolescent and young adult cancer survivors. Cancer.

[23] Stukaite-Ruibiene E., van der Perk M.E.M., Vaitkeviciene G.E., Bos A.M.E., Bumbuliene Z., Heuvel-Eibrink M.M.v.D., Rascon J. (2023). Evaluation of oncofertility care in childhood cancer patients: The EU-Horizon 2020 twinning project TREL initiative. Front. Pediatr..

[24] Omesi L., Narayan A., Reinecke J., Schear R., Levine J. (2019). Financial Assistance for Fertility Preservation Among Adolescent and Young Adult Cancer Patients: A Utilization Review of the Sharing Hope/LIVESTRONG Fertility Financial Assistance Program. J. Adolesc. Young-Adult Oncol..

[25] Takeuchi E., Kato M., Wada S., Yoshida S., Shimizu C., Miyoshi Y. (2017). Physicians’ practice of discussing fertility preservation with cancer patients and the associated attitudes and barriers. Support. Care Cancer.

[26] Panagiotopoulou N., van Delft F.W., Hale J.P., Stewart J.A. (2017). Fertility Preservation Care for Children and Adolescents with Cancer: An Inquiry to Quantify Professionals’ Barriers. J. Adolesc. Young-Adult Oncol..

[27] Panagiotopoulou N., Ghuman N., Sandher R., Herbert M., Stewart J. (2018). Barriers and facilitators towards fertility preservation care for cancer patients: A meta-synthesis. Eur. J. Cancer Care.

[28] Tishelman A.C., Sutter M.E., Chen D., Sampson A., Nahata L., Kolbuck V.D., Quinn G.P. (2019). Health care provider perceptions of fertility preservation barriers and challenges with transgender patients and families: Qualitative responses to an international survey. J. Assist. Reprod. Genet..

[29] Wang Y., Anazodo A., Logan S. (2019). Systematic review of fertility preservation patient decision aids for cancer patients. Psycho-Oncology.

[30] Hart R.J. (2019). Optimizing the opportunity for female fertility preservation in a limited time-frame for patients with cancer using in vitro maturation and ovarian tissue cryopreservation. Fertil. Steril..

[31] Gamzatova Z., Komlichenko E., Kostareva A., Galagudza M., Ulrikh E., Zubareva T., Sheveleva T., Nezhentseva E., Kalinina E. (2014). Autotransplantation of cryopreserved ovarian tissue—Effective method of fertility preservation in cancer patients. Gynecol. Endocrinol..

[32] Dittrich R., Hackl J., Lotz L., Hoffmann I., Beckmann M.W. (2015). Pregnancies and live births after 20 transplantations of cryopreserved ovarian tissue in a single center. Fertil. Steril..

[33] Hoover S.M., Bratton S.L., Roach E.R., Olson L.M. (2014). Parental experiences and recommendations in donation after circulatory determination of death. Pediatr. Crit. Care Med..

[34] Sque M., Walker W., Long-Sutehall T., Morgan M., Randhawa G., Rodney A. (2018). Bereaved donor families’ experiences of organ and tissue donation, and perceived influences on their decision making. J. Crit. Care.

[35] Luberda K., Cleaver K. (2017). How modifiable factors influence parental decision-making about organ donation. Nurs. Child. Young-People.

[36] Achkar T., Wilson J., Simon J., Rosenzweig M., Puhalla S. (2016). Metastatic breast cancer patients: Attitudes toward tissue donation for rapid autopsy. Breast Cancer Res. Treat..

[37] Frederick N.N., Klosky J.L., Meacham L., Quinn G.P., Kelvin J.F., Cherven B., Freyer D.R., Dvorak C.C., Brackett J., Ahmed-Winston S. (2023). Fertility Preservation Practices at Pediatric Oncology Institutions in the United States: A Report from the Children’s Oncology Group. JCO Oncol. Pract..

